# Effects of low energy availability on female reproductive function

**DOI:** 10.1002/rmb2.12414

**Published:** 2021-09-20

**Authors:** Takeshi Iwasa, Saki Minato, Junki Imaizumi, Atsuko Yoshida, Takako Kawakita, Kanako Yoshida, Yuri Yamamoto

**Affiliations:** ^1^ Department of Obstetrics and Gynecology Graduate School of Biomedical Sciences Tokushima University Tokushima Japan

**Keywords:** GnRH, hypothalamus, kisspeptin, metabolism, nutrition

## Abstract

**Background:**

It is known that metabolic and nutritional disturbances induce reproductive dysfunction in females. The main cause of these alterations is reduced gonadotrophin‐releasing hormone (GnRH) secretion from the hypothalamus, and the underlying mechanisms have gradually been elucidated.

**Methods:**

The present review summarizes current knowledge about the effects of nutrition/metabolism on reproductive functions, especially focusing on the GnRH regulation system.

**Main findings:**

Various central and peripheral factors are involved in the regulation of GnRH secretion, and alterations in their activity combine to affect GnRH neurons. Satiety‐related factors, i.e., leptin, insulin, and alpha‐melanocyte‐stimulating hormone, directly and indirectly stimulate GnRH secretion, whereas orexigenic factors, i.e., neuropeptide Y, Agouti‐related protein, orexin, and ghrelin, attenuate GnRH secretion. In addition, kisspeptin, which is a potent positive regulator of GnRH, expression is reduced by metabolic and nutritional disturbances.

**Conclusion:**

These neuroendocrine systems may be defensive mechanisms, which help organisms to survive adverse conditions by temporarily suppressing reproduction.

## 1 INTRODUCTION

It is known that metabolic and nutritional disturbances have various negative health consequences in females. In addition, it has been well established that reproductive functions are particularly susceptible to metabolic and nutritional status^1^, ^2^, ^3^, ^4^ and that the hypothalamus, which is located on the undersurface of the brain, plays pivotal roles in these interactions between reproduction and metabolism/nutrition.^5^ A negative energy status, which can be caused by eating disorders, weight loss due to calorie restriction, or excessive exercise, etc., frequently induces ovulatory disorders and/or irregular menses or amenorrhea and can disrupt sexual maturation.^1^, ^2^, ^3^, ^4^ It can also induce reductions in bone mineral density and increase the risk of osteoporosis due to the estrogen deficiency.^6^, ^7^, ^8^, ^9^ In addition, disturbances of energy utilization, such as obesity and diabetes, can also cause reproductive dysfunction, even though enough energy is stored in these conditions.^10^, ^11^, ^12^, ^13^

Although it has long been known that metabolic and nutritional status affects reproductive functions, the mechanisms underlying these effects were not revealed until the 1970s. In 1971, the structure of gonadotrophin‐releasing hormone (GnRH) was identified,^14^, ^15^ and the reproductive roles of GnRH, the mode of secretion of GnRH (pulses and surges), and the regulation of GnRH by gonadal hormones and the central nervous system were all clarified. In accordance with these advances in the field of reproductive endocrinology, the mechanisms underlying the effects of metabolic and nutritional status on reproductive functions have been vigorously evaluated in clinical and experimental studies. During the 1980s and early 1990s, it was revealed that decreased GnRH secretion from the hypothalamus is the main cause of the reproductive dysfunction induced by metabolic and nutritional disturbances.^16^, ^17^, ^18^, ^19^ During the 1990s and early 2000s, it was clarified that some peripheral and hypothalamic factors involved in the regulation of appetite and metabolism also regulate GnRH secretion and that changes in their activity due to under‐ or overnutrition may suppress GnRH secretion and cause concomitant modulation of feeding behavior.^20^, ^21^, ^22^, ^23^, ^24^ In 2003, it was discovered that kisspeptin and its receptor Kiss1r are expressed in the hypothalamus and that kisspeptin/Kiss1r signaling is a major stimulus for the secretion of GnRH.^25^, ^26^ Subsequent studies revealed that kisspeptin may be the missing link between sex steroid feedback activity and GnRH.^27^ Similarly, other studies have shown that kisspeptin activity is decreased by both under‐ and overnutrition, indicating that kisspeptin may also be involved in the reproductive dysfunction induced by metabolic and nutritional disturbances.^28^, ^29^ The aim of this review is to summarize current knowledge regarding the effects of metabolism/nutrition on reproductive functions and the neuroendocrine mechanisms underlying these effects, focusing, in particular, on the effects of a negative energy balance.

## 2 CONCEPT OF BRAIN ENERGY AVAILABILITY

**FIGURE 1:**
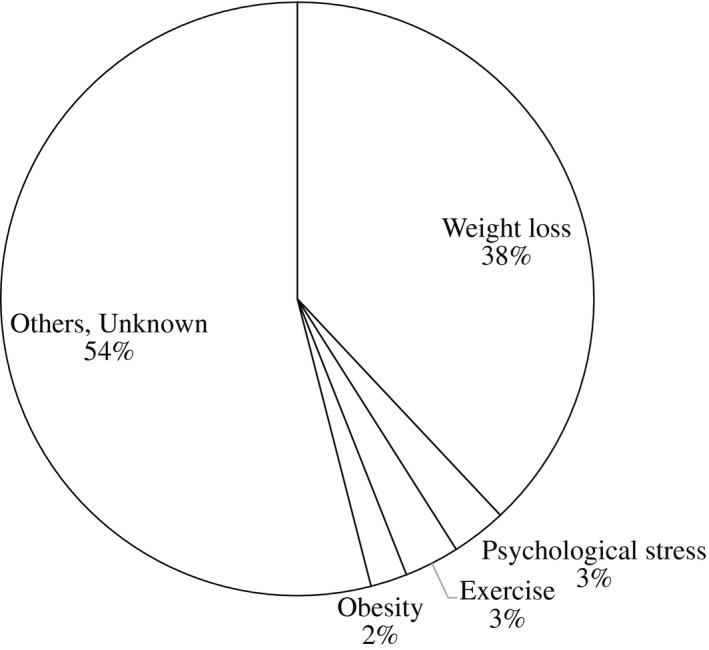
The causes of irregular menses or secondary amenorrhea associated with the hypothalamic‐pituitary system (adapted from ref 33)

In the 1970s and early 1980s, it was reported that the reproductive functions of females were disrupted by a lack of energy or a large energy drain. For example, the onset of puberty was markedly delayed in dancers who continued undergoing physical training involving a large energy drain.^30^ Although their sexual development and menarche were promoted when their exercise schedule was decreased and/or they were forced to rest due to injury, an amenorrheic state recurred after they returned to their original exercise schedule. Similarly, menstrual dysfunction or amenorrhea was induced in athletes, and these changes were related to a decreased body fat percentage.^31^, ^32^ In addition, our study evaluating the causes of irregular menses or secondary amenorrhea associated with the hypothalamic‐pituitary system indicated that 38% of cases were induced by body weight loss, and 3% were induced by psychological stress or exercise^33^ (Figure 1). As it had long been speculated that these effects of body weight loss and exercise on reproductive functions were induced by changes in hypothalamic function, the concept of brain energy availability was proposed in 1984.^34^ Namely, the brain appears to monitor the balance between the availability of calories and their utilization, and reproductive functions are transiently suppressed when the balance is unfavorable in order to survive such conditions.

## 3 THE EFFECTS OF LOW ENERGY AVAILABILITY ON GNRH

As noted above, although it has long been known that metabolic and nutritional status affects reproductive functions, the mechanisms underlying these effects were not revealed until the 1970s. In 1948, Harris proposed the hypothesis that hypothalamic neurons may secrete some neurohormones into the hypophyseal portal vein to regulate the levels of anterior pituitary hormones.^35^ McCann and Ramirez demonstrated the biological existence of GnRH (referred to as luteinizing hormone (LH)‐releasing factor), and Guillemin and Schally subsequently identified the structure of GnRH in 1971.^14^, ^15^ Further studies revealed that reproductive functions are mainly regulated by the hypothalamic‐pituitary‐gonadal (HPG) axis, i.e., the GnRH‐gonadotrophins‐gonadal steroids axis, in humans and animals. Among these factors, hypothalamic GnRH acts as a central regulator of the HPG axis, and it also plays pivotal roles in brain energy availability. Previous studies have shown that reductions in energy availability suppress HPG activity by inhibiting GnRH, thereby decreasing gonadotrophin secretion from the pituitary gland. As the secretion of GnRH from hypothalamic neurons into the hypophyseal portal vein is difficult to measure, most of these studies measured serum LH levels as an index of GnRH secretion, i.e., the pulsatile secretion of LH reflects the pulsatile secretion of GnRH, and the surge secretion of LH reflects GnRH surge secretion. The mean plasma LH levels of females with hypothalamic amenorrhea, whose symptoms were mainly caused by weight loss, were lower than those of normal females, and the LH pulse frequency was lower in females with hypothalamic amenorrhea than in normal females during the early follicular phase.^16^ In addition, it was found that LH pulse frequency does not decrease linearly along with energy status, but rather is disrupted when energy availability falls below a threshold level.^19^ Furthermore, a study evaluating GnRH secretion into the hypophyseal portal vein in female sheep revealed that both the frequency and amplitude of GnRH pulses were decreased in growth‐restricted hypogonadotropic sheep,^36^ and the pulsatile administration of GnRH induced ovulation in patients with hypothalamic amenorrhea,^37^, ^38^ supporting the hypothesis that a reduction in pulsatile GnRH secretion is the main cause of the reproductive dysfunction induced by a negative energy balance.

## 4 THE ROLES OF OREXIGENIC AND ANOREXIGENIC FACTORS IN THE REGULATION OF GNRH UNDER LOW ENERGY AVAILABILITY

**FIGURE 2:**
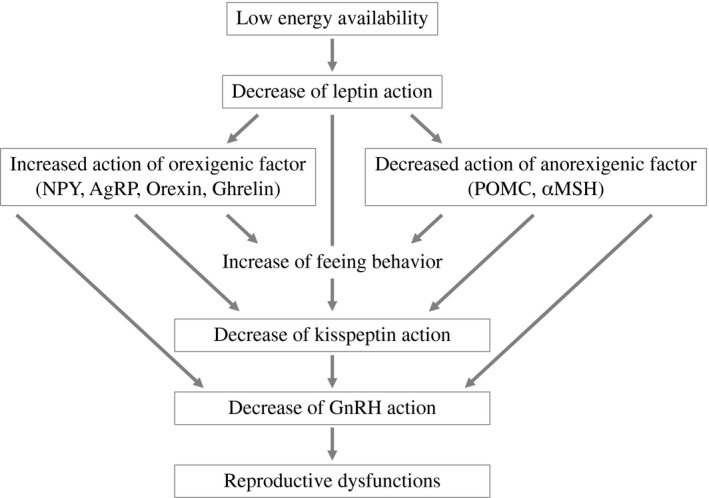
Hypothesis regarding the mechanisms responsible for the reproductive dysfunction seen under low energy availability conditions

As mentioned above, the reproductive dysfunction associated with a negative energy status is mainly induced by decreased GnRH pulsatile secretion. These alterations can be reproduced in some experimental animal models by energy restriction, and hence, such animal models have been used to investigate the neuroendocrine and hormonal mechanisms underlying these phenomena. As a result, it has been revealed that some appetite‐ or metabolism‐regulating factors affect GnRH neurons and that changes in their activity may suppress pulsatile GnRH secretion in the presence of under‐ or overnutrition (Figure 2). Generally, satiety‐related factors, such as leptin, insulin, alpha‐melanocyte‐stimulating hormone (αMSH), and proopiomelanocortin (POMC), have direct and indirect facilitative effects on GnRH, whereas orexigenic factors, such as neuropeptide Y (NPY), Agouti‐related peptide (AgRP), orexin, and ghrelin, have suppressive effects on GnRH.^20^, ^21^, ^22^, ^23^, ^24^ In low energy availability conditions, the activity levels of satiety‐related factors are decreased and those of orexigenic factors are increased, and consequently GnRH secretion is decreased. The detailed effects of each factor on GnRH neurons are described below.

### 4.1 Leptin

Leptin is an adipocyte‐derived satiety‐controlling peptide, and it plays pivotal roles in the regulation of appetite and reproduction. Leptin modulates appetite and metabolic rates through hypothalamic orexigenic and anorexigenic factors and prevents excessive weight gain and obesity in humans and animals.^39^, ^40^, ^41^ In addition to these effects on nutrition, leptin plays pivotal roles in sexual maturation and fertility in adulthood. Leptin‐deficient *ob*/*ob* mice exhibit disturbed sexual maturation and infertility due to low gonadotrophin levels, and chronic leptin treatment increased their serum gonadotrophin levels and restored puberty and fertility.^42^, ^43^ In addition, chronic leptin administration accelerated the onset of puberty in normally nourished female mice,^44^ and it also normalized serum gonadotrophin levels and restored estrous cyclicity in undernourished adult female mice.^45^ Although the facilitative effects of leptin on gonadotrophins are primarily mediated through the stimulation of GnRH neurons, GnRH neurons themselves do not express leptin receptors.^46^ In addition, the ablation of the leptin receptor from all forebrain neurons prevented the onset of puberty and induced infertility in male and female mice, indicating that leptin may indirectly act on GnRH neurons through some other forebrain factors.^47^

### 4.2 Insulin

It has been reported that insulin is also involved in the regulation of GnRH secretion. Neuron‐specific disruption of the insulin receptor (IR) gene induced a reduction in serum LH levels followed by hypogonadism in female mice.^48^ On the contrary, both male and female mice that were subjected to selective ablation of the IR from GnRH neurons displayed normal pubertal timing and fertility.^49^ These findings indicate that insulin also indirectly influences GnRH neurons to regulate reproductive functions. Interestingly, mice that were subjected to the deletion of insulin‐like growth factor 1 (IGF1) showed low gonadotrophin levels and delayed pubertal development, indicating that IGF1 may directly affect GnRH neurons.^49^

### 4.3 POMC and αMSH

Proopiomelanocortin is a precursor protein, which is used to produce biologically active peptides. POMC neurons within the hypothalamic arcuate nucleus (ARC) play a critical mediating role in leptin and insulin signaling and act as a vital anorexigenic factor.^50^ POMC neurons project into the medial preoptic area (POA), where GnRH neurons are concentrated, and some of them make a synaptic contact with GnRH neurons.^51^ αMSH is one of the cleavage products of POMC, and it acts as an anorexigenic neuropeptide by binding to the melanocortin 4 receptor (MC4R).^52^ GnRH neurons express MC4R, and the central administration of αMSH increased the serum LH levels of mice and rats.^53^, ^54^ In addition, MC4R‐deficient mice exhibited a decreased ovulation rate, and the normalization of melanocortin‐signaling ameliorated subfertility in leptin receptor knockout *db*/*db* mice.^54^, ^55^ These findings suggest that αMSH mediates leptin activity and directly stimulates GnRH secretion.

### 4.4 NPY, AgRP, orexin, and ghrelin

Neuropeptide Y, AgRP, orexin, and ghrelin are hypothalamic orexigenic factors. NPY neurons come into close contact with GnRH neurons and directly signal into GnRH neuron cell bodies and nerve terminals via the NPY Y1 receptor.^56^ Food deprivation increases hypothalamic NPY neuronal activity and mRNA expression, and concomitantly decreases LH secretion.^57^ In addition, the administration of NPY reduced gonadotrophin levels in female rats,^58^, ^59^ whereas gonadotrophin levels were not affected by fasting in NPY‐deficient mice.^60^ Furthermore, although obesity and infertility are seen in leptin‐deficient *ob*/*ob* mice, these phenotypes are ameliorated in NPY‐deficient *ob*/*ob* mice, suggesting that NPY functions as a central effector that mediates the effects of leptin on the appetite and reproductive systems.^61^ AgRP, which is a hypothalamic orexigenic factor, is co‐expressed with NPY in the neuronal population found in the ARC and is negatively regulated by leptin. It has been shown that AgRP has inhibitory effects on LH secretion in monkeys, and the ablation of AgRP‐expressing neurons in *ob*/*ob* mice restored their fertility.^62^, ^63^ These findings indicate that AgRP is also involved in the central effects of leptin deficiency. Orexin, which is produced by hypothalamic neurons, is involved in the control of appetite and arousal. Orexin neuron cell bodies are located in the lateral hypothalamus, and their fibers project into various areas of the brain, including the POA and ARC, where GnRH neurons are concentrated.^64^, ^65^ In addition, approximately 80% of GnRH neuron cell bodies express orexin receptors in rats, and orexin suppresses GnRH neuron activity in mice.^66^, ^67^ In previous studies, we showed that the intracerebroventricular injection of orexin decreased the GnRH pulse frequency and that these effects were partially mediated by β‐endorphin and corticotropin‐releasing hormone receptors.^21^, ^22^, ^23^ These results indicate that orexin has direct and indirect suppressive effects on GnRH neurons and that it might play a role in reducing GnRH secretion in low energy availability conditions. Ghrelin, which is found in endocrine cells in the gastric submucosa and the hypothalamic ARC, facilitates growth hormone secretion and promotes feeding behavior during fasting.^68^, ^69^ As is the case for other orexigenic factors, it has been shown that ghrelin also suppresses GnRH secretion in many species. The central or peripheral administration of ghrelin caused reductions in the GnRH pulse frequency and serum LH levels in rats, sheep, monkeys, and humans,^70^, ^71^, ^72^, ^73^, ^74^ and ghrelin also suppressed the release of GnRH from hypothalamic explants from rats.^75^ In addition, we previously showed that these effects of ghrelin on GnRH are partially mediated by β‐endorphin and NPY.^24^, ^76^

## 5 THE ROLES OF KISSPEPTIN IN THE REGULATION OF GNRH UNDER LOW ENERGY AVAILABILITY

Kisspeptin is a hypothalamic peptide, which directly stimulates the release and synthesis of GnRH through its receptor Kiss1r.^25^, ^26^, ^77^, ^78^, ^79^, ^80^ Kisspeptin neurons are concentrated in the ARC and anteroventricular periventricular nucleus (AVPV) in several species and are considered to mediate feedback signaling by estrogen. Namely, kisspeptin in the ARC mediates negative feedback from estrogen, whereas kisspeptin in the AVPV mediates positive feedback from estrogen.^27^ In addition to these physiological roles, it has been suggested that kisspeptin plays some pathophysiological roles in the reproductive dysfunction induced by negative energy availability (Figure 2).

### 5.1 The effects of low energy availability on hypothalamic kisspeptin signaling

Several studies have shown that kisspeptin is highly sensitive to metabolic and nutritional status, i.e., a negative energy balance had a negative impact on hypothalamic kisspeptin levels in rodents of various ages.^29^, ^81^, ^82^, ^83^, ^84^ Undernutrition reduced the hypothalamic expression of the *Kiss1* gene, which encodes kisspeptin, and disturbed sexual maturation and the onset of puberty in prepubertal female rats; however, the administration of exogenous kisspeptin normalized gonadotrophin secretion and the timing of puberty.^85^ Similarly, acute fasting disturbed estrous cyclicity and caused concomitant reductions in *Kiss1* mRNA expression and gonadotrophin levels.^83^ Some studies have shown that the effects of a negative energy balance on kisspeptin are different in each hypothalamic nucleus. For example, fasting reduced *Kiss1* mRNA expression in the ARC in gonadally intact female rats,^83^ whereas it reduced *Kiss1* mRNA expression in the AVPV in ovariectomized female rats.^86^ Similarly, *Kiss1* mRNA expression in the ARC was lower in lean ovariectomized ewes than normal‐weight ewes,^87^ and the number of kisspeptin immunoreactive neurons in the ARC was also lower in fasted lambs than in fed lambs.^88^ Interestingly, it has been reported that disturbances of energy utilization, such as obesity and diabetes, also affect the hypothalamic kisspeptin‐Kiss1r system. For example, hypothalamic *Kiss1* mRNA expression and gonadotropin levels were reduced in streptozotocin‐induced diabetic male rats, and the administration of kisspeptin restored normal serum gonadotropin levels.^89^ Similarly, high‐fat‐diet‐induced obesity reduced hypothalamic *Kiss1* mRNA expression and caused infertility in female mice.^90^ These findings indicate that the kisspeptin expressed on GnRH neurons integrates a range of metabolic inputs.

### 5.2 Mechanisms underlying the effects of low energy availability on kisspeptin

Although the exact mechanisms underlying the effects of low energy availability on kisspeptin remain unclear, it has been suggested that leptin, AgRP, and NPY might affect the neuronal activity of kisspeptin (Figure 2). It has been established that leptin acts as a positive regulator of GnRH neurons in many species; however, the leptin receptor is not expressed by GnRH neurons.^46^ Regarding this contradiction, some studies have suggested that hypothalamic kisspeptin may mediate the stimulatory effects of leptin on GnRH neurons. Namely, kisspeptin neurons in the ARC express the leptin receptor,^91^, ^92^ and the downregulation of leptin activity reduced hypothalamic *Kiss1* mRNA expression in mice and monkeys.^93^, ^94^ In addition, hypothalamic *Kiss1* mRNA expression is reduced in diabetic rats and *ob*/*ob* mice, but the administration of leptin restores normal *Kiss1* mRNA expression levels.^89^, ^90^ These findings support the hypothesis that the reductions in leptin levels induced by low energy availability suppress the effects of kisspeptin on GnRH, and these alterations may consequently induce reproductive dysfunctions. On the contrary, it has been shown that the deletion of the leptin receptor from hypothalamic kisspeptin neurons did not have any effect on sexual maturity or fertility in mice,^95^ indicating that the effects of kisspeptin on reproductive functions might not mediated by leptin. Thus, we should be aware that the relationship between leptin and kisspeptin has not been fully clarified. As is the case for leptin, AgRP and NPY are also considered to be implicated in the regulation of kisspeptin. Inhibitory synaptic connections exist between AgRP and kisspeptin neurons, and kisspeptin neurons received less marked presynaptic suppression when AgRP neurons were ablated.^96^, ^97^ In addition, kisspeptin neurons express NPY receptors; however, the neuroendocrine interactions between kisspeptin and NPY have not been clarified.^96^ These findings indicate that AgRP and NPY may suppress GnRH secretion and subsequently reduce fertility, and that kisspeptin may, at least in part, mediate these effects of AgRP and NPY.

### 5.3 The effects of overnutrition on hypothalamic kisspeptin signaling

As is seen in low energy availability conditions, kisspeptin activity is also reduced by overnutrition. Hypothalamic *Kiss1* mRNA expression is reduced in diet‐induced obese female mice; nevertheless, the serum leptin levels of these mice are elevated.^90^ In addition, the administration of leptin did not activate the leptin‐signaling molecules phosphorylated signal transducer and activator of transcription 3 (pSTAT3), pSTAT5, and phosphorylated ribosomal protein S6 in AVPV kisspeptin neurons in these animals, indicating that diet‐induced obesity may induce leptin resistance affecting central reproductive functions.^90^ Similarly, a recent study has shown that *Kiss1* mRNA expression in the ARC was reduced in diet‐induced obese rats and suggested that this alteration may be the initial pathological change in hypogonadotropic hypogonadism in these animals.^13^

## 6 CONCLUSION

Reproductive functions are affected by metabolic and nutritional conditions, and the suppression of GnRH secretion is the main cause of these impairments. Central and peripheral factors, such as appetite‐regulating factors and kisspeptin, are involved in the regulation of GnRH secretion, and alterations in their activity combine to affect GnRH neurons. These neuroendocrine systems may be defensive mechanisms that help organisms survive adverse conditions through the temporary suppression of reproduction. Recently, the number of couples who use assisted reproductive technology has been increased year by year. Although advanced techniques, such as IVF/ICSI, might be able to overcome the effects of metabolic and nutritional disorders, it is also important to remember that improvement of metabolic and nutritional conditions is needed in terms of preconception care.^98^, ^99^

## CONFLICT OF INTEREST

The authors declare that no conflicts of interest exist in this review. This article does not contain any studies with human and animal subjects performed by any of the authors.
